# Temporal changes in the associations between diagnosed psychiatric disorders and dropping out of school early

**DOI:** 10.1007/s00787-023-02252-2

**Published:** 2023-06-29

**Authors:** Ida Ringbom, Jaana Suvisaari, Andre Sourander, Mika Gissler, David Gyllenberg

**Affiliations:** 1 Department of Child Psychiatry and Invest Flagship, University of Turku, Lemminkäisenkatu 3, 3rd. Floor, 20014 Turku, Finland; 2 Finnish Institute for Health and Welfare, Helsinki, Finland; 3 Department of Adolescent Psychiatry, Helsinki University Hospital, Helsinki, Finland; 4 Department of Child Psychiatry, Turku University Central Hospital, Turku, Finland; 5 Department of Molecular Medicine and Surgery, Sweden and Region Stockholm, Academic Primary Health Care Centre, Karolinska Institute, Stockholm, Sweden

**Keywords:** Adolescence, Psychiatric disorders, School dropout, Epidemiology

## Abstract

Psychiatric problems are risk markers for poor educational attainment. The number of adolescents receiving treatment has increased. We investigated whether the association between psychiatric problems in early adolescence and dropping out of school had changed. We used the register-based 1987 and 1997 Finnish Birth Cohort studies, which include all live births in Finland. Hospital districts with incomplete records were excluded, leaving 25,421 participants born in 1987 and 32,025 born in 1997. The main outcome was not having applied for secondary education by the year the cohort members turned 18. Our main predictors were psychiatric and neurodevelopmental disorders diagnosed by specialized services during 1998–2003 and 2008–2013, when the cohort members were 10–16 years old. We found that 511 (2.0) of subjects born in 1987 and 499 (1.6%) born in 1997 dropped out of school. Having any diagnosis at 10–16 of age was associated with dropping out of school early in both cohorts: 3.9% in 1987 and 4.8% in 1997. The highest proportions were in the subgroup with autism spectrum disorders (ASD), 19.4% in 1987 and 16.2% in 1997. Dropping out early increased among adolescents diagnosed with any psychiatric or neurodevelopmental disorder, from 3.9 to 4.8%, with the clearest increase for learning disabilities, from 3.4 to 9.0%. Dropping out decreased for those with depression, from 4.5 to 2.1%. Adolescents with psychiatric and especially neurodevelopmental disorders, need effective interventions to prevent them dropping out of school early. Increased detection of psychopathology did not result in decreased dropout rates.

**Supplementary Information:**

The online version contains supplementary material available at 10.1007/s00787-023-02252-2.

## Introduction

Psychiatric problems in adolescence have long been regarded as a robust risk marker for poor educational attainment in both high-income and middle-income countries [1–8]. Understanding the paths to poor educational attainment is important, as they have been associated with reduced income, poor health, disability and mortality [9]. Most importantly, dropping out of school early has been shown to be a risk marker for marginalization and other adverse labour market outcomes. These have been a concern for policy makers in many countries [10, 11]. Given recent increases in treatment rates for psychiatric disorders [12], it is vital to identify temporal changes in the association between these problems during adolescence and educational attainment.

No clear increase in self-reported psychiatric symptoms were reported before the COVID-19 pandemic, although there might have been variations between different types of symptoms [13–16]. However, an increase in diagnosed psychiatric issues during adolescence has been reported [12, 17–19]. While there is no evidence of an increase in youth being marginalized in the labour market [11], we do not know how these changes have affected the associations between psychiatric and neurodevelopmental disorders and educational attainment. Furthermore, many of the studies about these associations used high-school graduation or college enrolment during early adulthood as the outcome measures and have not looked at even earlier problems in education [4, 6]. If it is possible to recognise risk markers for dropping out of school earlier, there will be a longer time span, when support and rehabilitation can be provided. This could increase the potential of interventions to prevent dropping out from school.

The main objective of this study was to establish how common it is for young people with psychiatric disorders to drop out of school already before starting upper secondary school education and if there has been changes in the associations as an increase in diagnosed psychiatric disorders has been reported. During the study periods, Finnish education consisted of 9 years of comprehensive basic education, followed by upper secondary school, where students could obtain a vocational diploma or focus on theoretical subjects. Adolescents usually finished compulsory education the year they turned 16 and during the same year they applied for, and started, upper secondary education. We studied adolescents who dropped out of school at this stage by using two nationwide birth cohorts, in 1987 and 1997.

## Methods

### Study design

We used data from the 1987 and 1997 Finnish Birth Cohort longitudinal studies. These are managed by the Finnish Institute for Health and Welfare and contain data from Finnish nationwide registers for all children born in Finland during those years. The studies were approved by the Institute’s review board and we obtained permission from the registered keepers to use the data sources, as required by Finnish law. All the data were pseudonymised and handled according to Finnish data protection legislation and regulations. Informed consent was not required, because none of the registered individuals were contacted.

The population-based cohorts comprised 59,476 individuals who were born in Finland in 1987 and 58,802 who were born in 1997. We excluded individuals who had lived outside Finland, had died before the end of the follow-up period or had been diagnosed with an intellectual disability. It is known that the reporting of outpatient visits in some hospital districts during the early study period were incomplete. Consistent with previous studies [12], incompleteness was defined as a clearly erroneous number of reported yearly visits, which was found in 12 of 21 hospital districts (see Supplemental Table 1). To ensure that changes in service use were not biased by incomplete reporting we excluded individuals who resided in any of the hospital districts with incomplete data. The final cohort comprised 25,421 participants born in 1987 and 32,025 born in 1997. They represented 42.7% and 54.5% of the respective live births. We followed the participants until the year when they reached 18 years of age: those born in 1987 were followed until the end of 2005 and those born in 1997 until the end of 2015.

### Description of the registers

Data from six registers were linked to each individual using the unique personal identification code that is assigned to Finnish citizens and permanent residents. The Medical Birth Register identified the subjects’ mothers. The Digital and Population Data Services Agency provided emigration data and identified the subjects’ fathers. Statistics Finland provided the parents’ education levels and details on when any cohort members had died. Data on welfare benefits and out-of-home placements by child protection authorities were provided by the Finnish Institute for Health and Welfare. The Joint Application Register provided information on whether the subjects had applied for upper secondary education or not. These registers have previously been described in detail [20, 21].

Information on diagnoses were obtained from the Finnish Care Register for Health Care, which is maintained by the Finnish Institute for Health and Welfare. These were recorded during visits to physicians at inpatient or outpatient clinics in hospitals and specialized health care services across Finland. The records are regularly submitted to the Register by the hospitals and hospital districts. The Register includes the start and end dates of the visits, a mandatory primary diagnosis and optional secondary diagnoses. It has contained inpatient data since 1967 and outpatient data since 1998. However, as mentioned earlier, several hospital districts had incomplete reporting of outpatient care during the first years of specialized outpatient care registration, and the outpatient care register is complete only from 2006 onwards. This Register has been widely used for epidemiological research [21] and the diagnostic validity of the mental disorders in the Register has been studied for conditions such as autism [22].

### Definitions of outcomes

The main outcome was applying for upper secondary education. Most Finnish residents (99.7%) complete the nine-year basic education syllabus on time, but a voluntary tenth year was offered to those who needed support to progress to upper secondary education. Compulsory education was based on equality and inclusion. Children and adolescents studied in heterogeneous groups. Their socioeconomic background did not affect which schools they attended, because basic education was the same for everyone, schools were primarily selected by location and there were only small differences between schools. Every child was legally entitled to special needs education and student welfare support if they needed it [23]. Adolescents usually finished compulsory education the year they turned 16 and during the same year they applied for, and started, upper secondary education. The cohort members in this study turned 16 in 2003 and 2013 respectively. We counted the applications for the two following years as well, to allow for subjects who had started or finished their education later than expected. An adolescent was considered to have dropped out of school early if they did not apply for upper secondary education during this period.

### Predictors

The main predictors were psychiatric and neurodevelopmental disorders diagnosed by specialised services in 1998–2003 and 2008–2013 respectively, which was when the cohort members were 10–16 years old. The first start date was chosen because outpatient data were available from the Finnish Care Register for Health Care from 1998 onwards. Only inpatient data were available before that year. The end dates were chosen because we wanted to study disorders diagnosed by the time the cohort members were expected to finish their compulsory education. The diagnoses were recorded according to the International Statistical Classification of Diseases and Related Health Problems, Tenth Revision (ICD-10). They were categorised into diagnostic entities, in line with previous reports [24], and are described in detail (Supplemental Table 2). One person could have several different diagnoses.

Being placed in out-of-home care by child protection authorities by the age of 16, in 2003 and 2013 respectively, was used as a marker of mental ill-health and adversity. This was a secondary predictor [25].

### Socioeconomic background factors

The potential covariates were derived from registers, and we chose three background factors that had already been associated with children’s educational attainment: parental educational attainment, family structure [26] and parental socioeconomic status [27]. Parental education was divided into three categories and based on the highest category reached by either parent: just received compulsory education, completed upper secondary education and obtained a university degree. Family structure was split into having married parents or any other form of family. Welfare benefits were used as a measure of socioeconomic status. Information about ethnicity is not registered by the Finnish authorities.

### Statistical methods

We used logistic regression to carry out statistical modelling for the association between the disorders and educational attainment, due to the binary outcomes. First, we used univariate models to study one predictor at a time. Then, we examined the independent effects of the disorders by using multivariate models that comprised each of the diagnostic categories and the relevant covariates. To test the robustness of our findings, we investigated the effect of excluding hospital districts with reporting issues, by rerunning the analyses without district exclusions.

Additionally, we studied the association between dropping out of school in relation the number of diagnostic categories an individual had been diagnosed with disorders from; the same covariates were used in the multivariate model. We used the categorisation of diagnoses shown in Supplemental Table 2, with psychiatric diagnoses not falling into any of the mentioned categories forming their own category.

The standard error of the proportion was calculated, and the proportions reported with a 95% confidence interval. The additive interaction was calculated to study the change in the proportions of adolescents who drop out of school across the cohorts. This method calculates relative excess risk due to interaction [28].

R statistical software, version 3.4.0 (R Foundation, Vienna, Austria) was used for the analyses.

## Results

### Participants and setting

The 1987 Finnish Birth Cohort study comprised 59,476 people who were born in Finland and survived the perinatal period. We found that 323 (0.5%) died before the end of the follow-up period in 2003, when the subjects were 16 years of age. Another 806 had emigrated and 166 were diagnosed with an intellectual disability. A further 32,760 were excluded because they had been living in a hospital district with incomplete data. This means that 25,421 (53.6% male) subjects were included in the analysis, which represented 42.7% of the original 1987 birth cohort.

The 1997 cohort included 58,802 people. Of these 243 (0.4%) died before the end of the follow-up period in 2013, when the subjects were 16 years of age. Another 1320 had emigrated and 273 were diagnosed with an intellectual disability. A further 24,938 were excluded because they had been living in hospital districts with incomplete data. The final number included in the analyses was 32,025 (56.5% male), which was 54.5% of the original 1997 birth cohort.

### Descriptive results of diagnoses, service use and sociodemographic characteristics

We found that 2064 (8.1%) of those born in 1987 and 2440 (7.6%) of those born in 1997 had been diagnosed with a psychiatric or neurodevelopmental disorder when they were 10–16 years of age. The most common diagnoses in the 1987 cohort were learning disabilities, which affected 526. Anxiety disorders were the most common diagnoses in the 1997 cohort and 738 subjects were affected. The diagnostic category with the largest change in diagnoses was autism spectrum disorders, with three times as many diagnoses in 1997 than 1987: 229 (0.7%) versus 67 (0.3%). Of the members of the 1987 cohort 1778 had received diagnoses from one diagnostic category, 243 from two, 37 from three and six from four. In the 1997 cohort, 1954 had diagnoses from one category, 394 from two, 78 from three and 14 from four. No one had diagnoses from five or more categories in either cohort.

The sociodemographic characteristics of the sample are shown in Table 1. When we compared the two cohorts, we could see that the 1997 parents were more highly educated than the 1987 parents. This was measured using the highest level of education achieved by either parent and the percentage with a university degree had risen from 49.3 to 65.9%. We also found that at least one parent had received welfare support in 36.7% of cases in 1987 and this had risen to 41.5% by 1997. There was little difference in sociodemographic factors between the full 1987 cohort and the parents of those who had dropped out of school early. However, adolescents were more likely to drop out of school in the 1997 cohort if both parents had only completed compulsory education rather than gone on to upper secondary education or achieved a university degree (6.3 vs 1.3%).

**Table 1:** The prevalence of dropping out of school early, by sociodemographic characteristics and psychiatric and neurodevelopmental disorders

	1987		1997	
	Total n = 25,421	Dropped out n = 511	Total n = 32,025	Dropped out n = 499
Characteristic	n (%)	n (%)	n (%)	n (%)
Male	13,620 (53.6)	227 (44.4)	17,087 (53.4)	282 (56.5)
Parent received welfare support	9,340 (36.7)	173 (33.9)	13,283 (41.5)	201 (40.3)
Parents not married	12,823 (50.4)	257 (50.3)	13,603 (42.5)	262 (52.5)
Parental education				
Compulsory	2,188 (8.6)	49 (9.6)	651 (2.0)	41 (8.2)
Upper secondary	10,690 (42.1)	153 (29.9)	10,285 (32.1)	175 (35.1)
University	12,543 (49.3)	309 (60.5)	21,089 (65.9)	283 (56.7)
Out-of-home care by child protection	538 (2.1)	20 (3.9)	335 (1.0)	15 (3.0)
Any psychiatric or neurodevelopmental disorder	2,064 (8.1)	80 (15.7)	2,440 (7.6)	116 (23.2)
Depressive disorders	512 (2.0)	23 (4.5)	584 (1.8)	12 (2.4)
Anxiety disorders	502 (2.0)	21 (4.2)	738 (2.3)	23 (4.6)
Learning disabilites	526 (2.1)	18 (3.5)	591 (1.8)	53 (10.6)
Autism spectrum disorder	67 (0.3)	13 (2.5)	229 (0.7)	37 (7.4)
Conduct disorder	337 (1.3)	16 (3.1)	273 (0.9)	11 (2.2)

### Dropping out of school

The proportion of cohort members who had not applied for upper secondary education by the year when they turned 18 decreased slightly: 2.0% (95% CI 1.8–2.1) in the 1987 cohort and 1.6% (95% CI 1.4–1.7) in the 1997 cohort.

The proportion of the cohort members who had been diagnosed with a psychiatric or neurodevelopmental disorder who dropped out of school early rose from 3.9% (95% CI 3.0–4.7) in the 1987 cohort to 4.8% (95% CI 3.9–5.5) in the 1997 cohort. The p-value for the relative excess risk due to interaction was 0.01. The proportion was 4.6% (95% CI 3.4–5.9) among boys and 3.1% (95% CI 2.0–4.1) among girls in the 1987 cohort and 5.1% (95% CI 3.9–6.2) among boys and 4.2% (95% CI 3.0–5.4) among girls in the 1997 cohort. Among those without a diagnosis of a psychiatric or neurodevelopmental disorder 1.8% (95% CI 1.7–2.0) in the 1987 and 1.3% (95% CI 1.2–1.4) in the 1997 cohort dropped out.

**Fig. 1:**
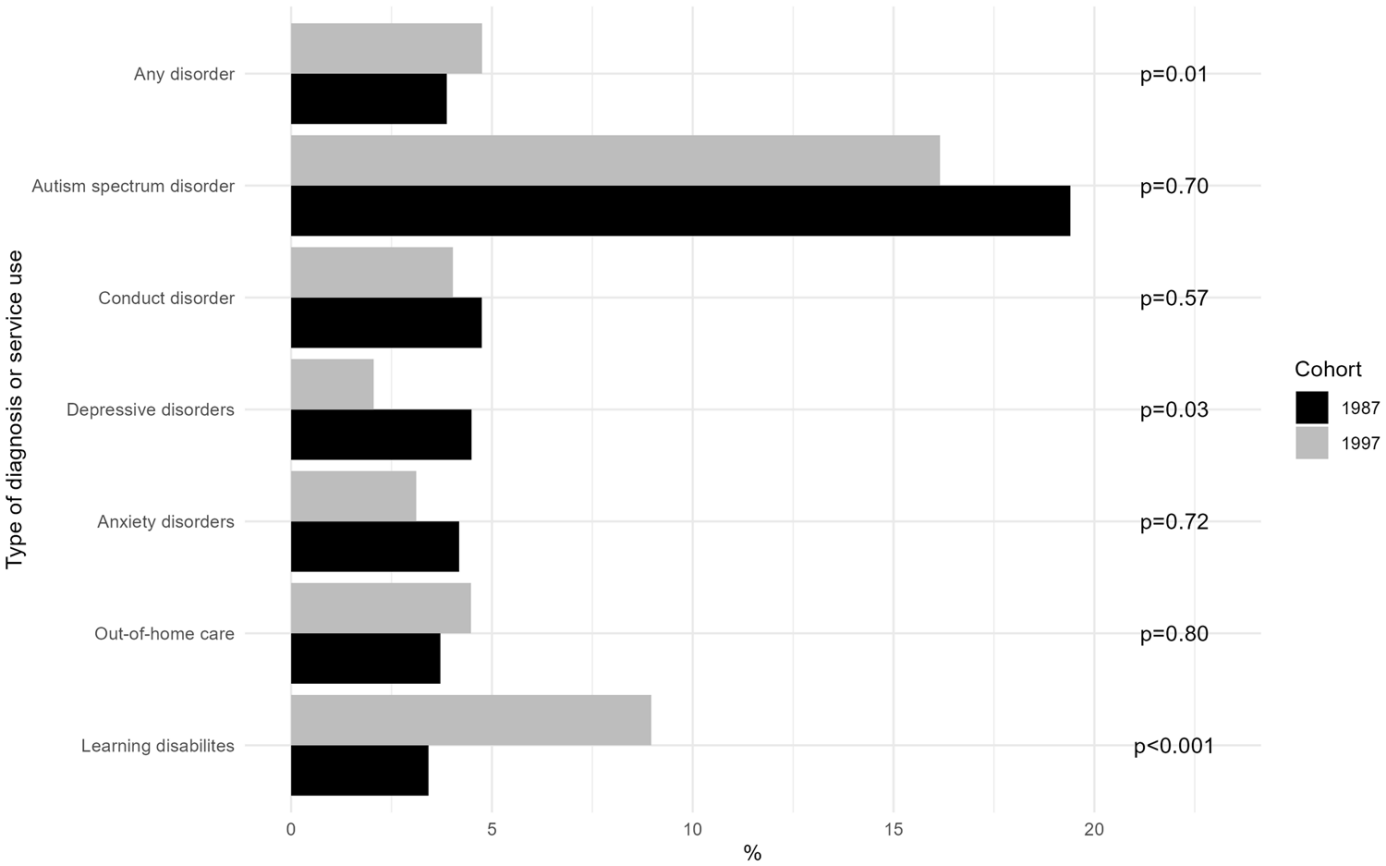
Subjects who dropped out of school early by diagnostic categories and placement in out-of-home care by child protection services. Presented as p values for the relative excess risk due to interaction

We found that 20 (3.7%, 95% CI 2.3–5.1) of the children in the 1987 cohort who had been placed in out-of-home care had subsequently dropped out of school early and 15 (4.5%, 95% CI 2.3–6.7) had dropped out in the 1997 cohort. The most common diagnoses among those who dropped out were depressive disorders in the 1987 cohort, 23, and learning disabilities in the 1997 cohort, 53. The diagnostic group with the largest proportion of adolescents who dropped out of school early was autism spectrum disorders: 13 (19.4%, 95% CI 9.9–28.9) in the 1987 cohort and 37 (16.2%, 95% CI 11.4–20.9) in the 1997 cohort (Fig. 1).

The largest change in the number of adolescents who dropped out of school early occurred in those with learning disabilities. There was no clear change in the proportion diagnosed from the 1987 and 1997 cohorts: 526 (2.1%, 95% CI 1.9–2.2) versus 591 (1.8%, 95% CI 1.7–2.0), but almost three times as many dropped out of school early in the 1997 than 1987 cohort: 53 (9.0%, 95% CI 6.7–11.3) versus 18 (3.5%, 95% CI 1.9–5.0). The p-value for the relative excess risk due to interaction was < 0.001.

We also saw a change in the number of adolescents with depression who dropped out of school early, which halved between 1987 and 1997 cohorts, from 23 (4.5%, 95% CI 2.7–6.3) to 12 (2.1%, 95% CI 0.9–3.2). The *p* value for the relative excess risk due to interaction was 0.03.

In the univariate analysis there was a significant association for all studied diagnostic categories but learning disorders and dropping out of school in the 1987 cohort and for all categories but depression in the 1997 cohort. Table 2 shows the multivariate analyses of the association between the different diagnostic categories and dropping out of school taking into account the following sociodemographic characteristics: sex, parental educational attainment, parental marital status, if at least one parent had received welfare benefits. In the 1987 cohort there were statistically significant associations between all the psychiatric diagnoses that were studied and adolescents who dropped out of school. In the 1997 cohort there were significant associations in the multivariate analyses for all diagnostic categories except for depressive disorders. The associations between any psychiatric or neurodevelopmental diagnosis and dropping out of school was stronger in the 1997 cohort than in the 1987 cohort in the multivariate analyses (OR 3.7 95% CI 2.9–4.5, p < 0.001 for 1997 and OR 2.2, 95% CI 1.7–2.8, p < 0.001 for 1987, p value for the interaction < 0.001). The strongest association in both cohorts in the multivariate analysis was between autism spectrum disorders and adolescents who dropped out of school (OR 13.5 95% CI 7.0–24.4, p < 0.001 for 1987 and, OR 12.4 95% CI 8.4–17.8, p < 0.001 for 1997). The associations were similar in the sensitive analysis including all hospital districts, see Supplemental Table 3.

**Table 2:** Multivariate analyses of the association between dropping out of school and psychiatric or neurodevelopmental diagnoses or out-of-home care by child protection in the 1987 and 1997 cohorts

	1987	1997
Diagnostic group	OR (95% CI), p value	OR (95% CI), p value
Any psychiatric or neurodevelopmental disorder	2.2 (1.7–2.8), p < 0.001	3.7 (2.9–4.5), p < 0.001
Depressive disorders	2.3 (1.5–3.5), p < 0.001	1.3 (0.7–2.2), p = 0.38
Anxiety disorders	2.1 (1.3–3.2), p = 0.001	2.0 (1.3–3.1), p = 0.01
Learning disabilites	2.0 (1.2–3.1), p = 0.005	6.3 (4.6–8.4), p < 0.001
Autism spectrum disorder	13.5 (7.0–24.4), p < 0.001	12.4 (8.4–17.8), p < 0.001
Conduct disorder	2.8 (1.6–4.5), p < 0.001	2.2 (1.1–3.9), p = 0.01
Out-of-home-care by child protection	2.2 (1.3–3.5), p = 0.001	2.0 (1.1–3.3), p = 0.02

*OR* odds ratio; *CI* confidence interval

The multivariate analysis included sex and parental sociodemographic characteristics

Of those in the 1987 cohort with diagnoses from one diagnostic category 61 (3.4%, 95% CI 2.6–4.3) dropped out from school and of those with diagnoses from two or more categories 19 (6.6%, 95% CI 3.8–9.5). In the 1997 of those with diagnoses from one category 88 (4.5%, 95% CI 3.6–5.4) dropped out and of those with diagnoses from two or more categories 28 (5.8%, 95% CI 3.7–7.8) dropped out. In the 1987 cohort the odds ratio in the multivariate analysis with the same sociodemographic factors for the association between dropping out of school and having diagnoses from one category was 2.0 (95% CI 1.5–2.6, p < 0.001) and for having two or more diagnoses was 4.0 (95% CI 2.4–6.4, p < 0.001). In the 1997 cohort the odds ratio for having diagnoses from one category was 3.5 (95% CI 2.7–4.4, p < 0.001) and from two or more categories 4.4 (95% CI 2.9–6.4, p < 0.001).

## Discussion

Our primary finding was that a significant proportion of those diagnosed with a psychiatric or neurodevelopmental disorder at 10–16 years of age dropped out of school early before they reached upper secondary education. The secondary finding was that the proportion of those diagnosed with any psychiatric or neurodevelopmental disorder who dropped out of school increased between 1987 and 1997 (3.9 vs 4.8%). The figures for the full cohort were 2.0% and 1.6%, respectively. As many as one in six of those diagnosed with autism spectrum disorder in both cohorts did not apply for upper secondary education. Our third finding was that the proportion of adolescents with learning disabilities who dropped out of school increased between the cohorts, while the proportion with depression decreased.

Although previous studies have shown an increase in diagnosed psychiatric disorders [12, 17–19], we did not see that for psychiatric and neurodevelopmental disorders in general in this study, which focused on diagnoses from 10 to 16 years of age. Still there was an increase in the number who dropped out of school among those who had any diagnosis of a psychiatric and neurodevelopmental disorders in general. There could be several explanations for this increase, and they could differ between diagnoses. This is discussed below.

The proportion of those diagnosed with autism spectrum disorders who dropped out did not change between the cohorts, even though the number of diagnosed cases more than tripled. Apparently, the increase in this diagnosis did not affect the educational outcomes in any way.

The proportion of those diagnosed with learning disabilities who dropped out of school early increased between the cohorts. Both cohorts contained more than 500 people who had been diagnosed with a learning disability, but there were three times as many who had dropped out of school early in 1997 than 1987: 53 versus 18. The pattern for depression was different, as the proportion who dropped out of school halved from 23 (4.5%) in the 1987 cohort to 12 (2.1%) in 1997. These changes could be due to changes in schools or healthcare systems or changes in wider society, such as urbanization, immigration and the economic crisis that started in 2007 [29].

Education in Finland changed during the first decades of the twenty-first century. A number of factors may have affected the support students with learning disabilities received in school during the study periods. The National Core Curriculum, which was launched in 1994, only gave very broad national guidelines. However, the national legislative and curricular norms were strengthened from 1998 to 2003 [23]. The economic crisis peaked globally during 2008 and significant cuts were made to Finnish school budgets in 2011 as a result [29]. According to one study, school burnout increased among students in their last years of compulsory education during 2006–2019, particularly after 2011 [29].

Some changes in psychiatric care for adolescents may have occurred during the same time, such as more referrals, more diverse care and increased use of psychotropic medication [13, 30]. These changes could have improved the outcomes of depression.

### Strengths and limitations

The main strength of this study was that it covered large, nationwide samples born in 1987 and 1997 and we were able to establish links between a large number of registers. The following limitations should be considered. The follow-up time was short, but we know that early educational problems predict later marginalisation [10]. The only educational data that were available were applications for upper secondary education. Only diagnoses made by specialised services could be analysed, which affected the sensitivity of the study. The diagnostic validity of autism spectrum disorders [22] in the register data has been good, but the diagnostic validity of all mental and neurodevelopmental disorders has not been studied. Outpatient data were available from 1998 onwards, which was when the children born in 1987 turned 11 years old. This means that we had high-quality data on disorders treated during adolescence, but not childhood, and the number of diagnosed neurodevelopmental disorders might have been smaller than it should have been. Only those individuals whose lives were still considerably affected by neurodevelopmental disorders in adolescence would have visited specialised care services by the time the follow-up data were collected. Although our study population was large, future studies with even larger sample sizes could allow for studying which combinations of diagnoses are associated with school dropout. About half of the Finnish hospital districts were not included in the main analyses, and thus our findings might not be representative for the whole country. However, the findings of the sensitivity analyses with all the hospital districts included were similar to those of the main analyses, suggesting that the generalisability is acceptable. Finally, exact generalisations to other countries should be avoided, due to differences in healthcare services, school systems and welfare benefits.

An important question for future studies is why children who had not been diagnosed with a psychiatric or neurodevelopmental disorder dropped out of school. Is there a group of adolescents who are not reached by the healthcare and child protection services, even though they need help? Other questions are why there was a stronger association between adolescents who dropped out of school and learning disabilities in the 1997 than 1987 cohort. Given the high predictive values a question for future intervention studies would be the cost-effectiveness of possible solutions for preventing dropping out from school.

## Conclusions

The study showed a strong association between adolescents who dropped out of school early and psychiatric and neurodevelopmental disorders. The findings that about one in six of those who had been diagnosed with an autism spectrum disorder in both cohorts did not apply for upper secondary education is alarming. Furthermore, those who had been diagnosed with a psychiatric or neurodevelopmental disorder, especially those with a learning disability were more likely to drop out early in the 1997 than the 1987 cohort. We need to ensure that neurodevelopmental disorders are diagnosed and that children and adolescents with these disorders receive support at school, and through other forms of rehabilitation, to enable them to finish their education.

### Supplementary Information

Below is the link to the electronic supplementary material.

Supplementary file1 (XLSX 14 KB)

## References

[1] Jackson M (2009). Understanding links between adolescent health and educational attainment. Demography.

[2] Lee S, Tsang A, Breslau J, Aguilar-Gaxiola S, Angermeyer M (2009). Mental disorders and termination of education in high-income and low- and middle-income countries: epidemiological study. Br J Psychiatry.

[3] Jonsson U, Bohman H, Hjern A (2010). Subsequent higher education after adolescent depression: a 15-year follow-up register study. Eur Psychiatry.

[4] Breslau J, Miller E, Joanie Chung W-J, Schweitzer JB (2011). Childhood and adolescent onset psychiatric disorders, substance use, and failure to graduate high school on time. J Psychiatr Res.

[5] McLeod JD, Uemura R, Rohrman S (2012). Adolescent mental health, behavior problems, and academic achievement. J Health Soc Behav.

[6] Esch P, Bocquet V, Pull C (2014). The downward spiral of mental disorders and educational attainment: a systematic review on early school leaving. BMC Psychiatry.

[7] Leadbeater BJ, Ames ME (2017). The longitudinal effects of oppositional defiant disorder symptoms on academic and occupational functioning in the transition to young adulthood. J Abnorm Child Psychol.

[8] Mikkonen J, Remes H, Moustgaard H, Martikainen P (2020). Evaluating the role of parental education and adolescent health problems in educational attainment. Demography.

[9] Zajacova A, Lawrence EM (2018). The relationship between education and health: reducing disparities through a contextual approach. Annu Rev Public Health.

[10] Myrskylä P. Hukassa – keitä ovat syrjäytyneet nuoret? (Lost – who are marginalised youth?) [In Finnish] *Elinkeinoelämän Valtuuskunnan analyysi* no 19. 2012.

[11] Eurofound. Exploring the diversity of NEETs. 2016. Luxembourg: Publications Office of the European Union

[12] Gyllenberg D, Marttila M, Sund R (2018). Temporal changes in the incidence of treated psychiatric and neurodevelopmental disorders during adolescence: an analysis of two national Finnish birth cohorts. Lancet Psychiatry.

[13] Mojtabai R, Olfson M, Han B (2016). National trends in the prevalence and treatment of depression in adolescents and young adults. Pediatrics.

[14] Mishina K, Tiiri E, Lempinen L (2018). Time trends of Finnish adolescents’ mental health and use of alcohol and cigarettes from 1998 to 2014. Eur Child Adolesc Psychiatry.

[15] Cosma A, Költő A, Badura P, Winkler P, Kalman M (2021). Time trends in adolescent mental wellbeing in the Czech Republic between 2002 and 2018: gender, age and socioeconomic differences. Cent Eur J Public Health.

[16] Knaappila N, Marttunen M, Fröjd S, Kaltiala R (2021). Changes over time in mental health symptoms among adolescents in Tampere, Finland. Scand J Child Adolesc Psychiatr Psychol.

[17] Atladottir HO, Gyllenberg D, Langridge A (2015). The increasing prevalence of reported diagnoses of childhood psychiatric disorders: a descriptive multinational comparison. Eur Child Adolesc Psychiatry.

[18] Comeau J, Georgiades K, Duncan L, Wang L, Boyle MH, Ontario Child Health Study Team (2014). Changes in the prevalence of child and youth mental disorders and perceived need for professional help between 1983 and 2014: evidence from the Ontario child health study. Can J Psychiatry.

[19] Russell G, Stapley S, Newlove-Delgado T, Salmon A, White R, Warren F, Pearson A, Ford T (2022). Time trends in autism diagnosis over 20 years: a UK population-based cohort study. J Child Psychol Psychiatry.

[20] Gissler M, Haukka J (2004). Finnish health and social welfare registers in epidemiological research. Nor Epidemiol.

[21] Sund R (2012). Quality of the Finnish Hospital discharge register: a systematic review. Scand J Public Health.

[22] Lampi KM, Sourander A, Gissler M (2010). Brief report: validity of Finnish registry-based diagnoses of autism with the ADI-R. Acta Paediatr.

[23] Järvinen, R. Current Trends in Inclusive Education in Finland. Finnish National Board of Education. 2007

[24] Ringbom I, Suvisaari J, Kääriälä A (2022). Psychiatric disorders diagnosed in adolescence and subsequent long-term exclusion from education, employment or training: longitudinal national birth cohort study. Br J Psychiatry.

[25] Kääriälä A, Gyllenberg D, Sund R (2022). The association between treated psychiatric and neurodevelopmental disorders and out-of-home care among Finnish children born in 1997. Eur Child Adolesc Psychiatry.

[26] d'Addio A (2007) Intergenerational transmission of disadvantage: mobility or immobility across generations? A review of the evidence for OECD Countries. OECD Social, employment and migration working papers

[27] Schulz W, Schunck R, Diewald M, Johnson W (2017). Pathways of intergenerational transmission of advantages during adolescence: social background, cognitive ability, and educational attainment. J Youth Adolesc.

[28] Zou GY (2008). On the estimation of additive interaction by use of the four-by-two table and beyond. Am J Epidemiol.

[29] Read S, Hietajärvi L, Salmela-Aro K (2022). School burnout trends and sociodemographic factors in Finland 2006–2019. Soc Psychiatry Psychiatr Epidemiol.

[30] Kronström K, Kuosmanen L, Ellilä H, Kaljonen A, Soundander A (2018). National time trend changes in psychotropic medication of child and adolescent psychiatric inpatients across Finland. Child Adolesc Ment Health.

